# Utilisation of podiatry services in Australia under the Medicare Enhanced Primary Care program, 2004-2008

**DOI:** 10.1186/1757-1146-2-30

**Published:** 2009-10-30

**Authors:** Hylton B Menz

**Affiliations:** 1 Musculoskeletal Research Centre, Faculty of Health Sciences, La Trobe University, Bundoora, Victoria 3086, Australia

## Abstract

**Background:**

In 2004, as an extension of the Enhanced Primary Care (EPC) program, the Australian Government introduced a policy of providing Medicare rebates for allied health services provided to patients with chronic or complex health conditions. The objective of this study was to evaluate the utilisation of podiatry services provided under this scheme between 2004 and 2008.

**Methods:**

Data pertaining to the Medicare item 10962 for the calendar years 2004-2008 were extracted from the Australian Medicare Benefits Schedule (MBS) database and cross-tabulated by sex and age. Descriptive analyses were undertaken to assess sex and age differences in the number of consultations provided and to assess for temporal trends over the five-year assessment period. The total cost to Medicare over this period was also determined.

**Results:**

During the 2004-2008 period, a total of 1,338,044 EPC consultations were provided by podiatrists in Australia. Females exhibited higher utilisation than males (63 versus 37%), and those aged over 65 years accounted for 75% of consultations. There was a marked increase in the number of consultations provided from 2004 to 2008, and the total cost of providing EPC podiatry services during this period was $62.9 M.

**Conclusion:**

Podiatry services have been extensively utilised under the EPC program by primary care patients, particularly older women, and the number of services provided has increased dramatically between 2004 and 2008. Further research is required to determine whether the EPC program enhances clinical outcomes compared to standard practice.

## Background

Management of chronic disease accounts for a considerable degree of healthcare expenditure in Australia, with recent data indicating that chronic medical conditions are responsible for more than 80% of the total burden of disease and injury [1]. In 1999, the Australian Government introduced the Enhanced Primary Care (EPC) program to improve the coordination of health care for people with chronic and complex conditions [2]. As part of an extension and redevelopment of this scheme, chronic disease management items were added to Medicare in 2004, enabling rebates to be paid for individual services provided by allied health professionals, including aboriginal health worker services, audiology, mental health services, psychology, occupational therapy, diabetes education, osteopathy, exercise physiology, speech pathology, chiropractic, dietetics, physiotherapy and podiatry [3].

**Figure 1:**
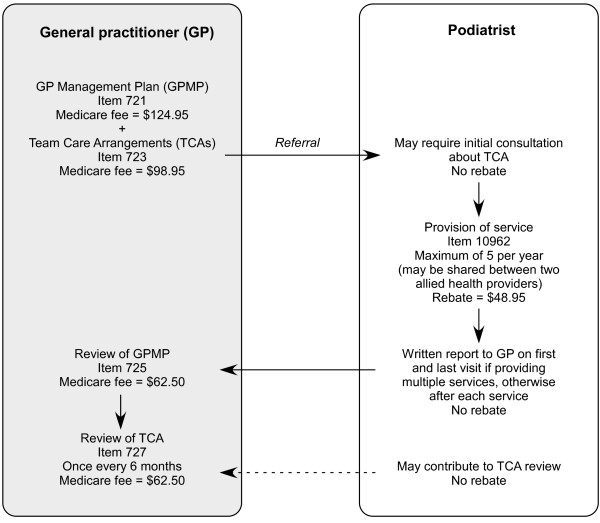
**Schematic representation of the EPC program**. Note that the system is not a GP fundholder model - the podiatrist is paid by the patient at the time of consultation and the patient is subsequently reimbursed by Medicare.

To be eligible for rebates, patients are required to have a chronic medical condition present for at least six months (such as asthma, cancer, cardiovascular disease, diabetes, mental disorders and arthritis or other musculoskeletal condition), or have complex care needs (defined as requiring ongoing care from their general practitioner [GP] and at least two other health care providers). The EPC chronic disease management program is coordinated by the patient's GP, who prepares a management plan, initiates the referrals to allied health professionals, and reviews progress every six months. A maximum of five allied health services is allowed per calendar year. Although allied health practitioners can set their own fees, each consultation attracts a maximum Medicare rebate of $48.95 [4]. A schematic representation of the EPC program (adapted from Foster et al [5]) is provided in Figure 1, and a full explanation of the program can be accessed at the Medicare website [4].

In a recent critique of the EPC program, Foster et al [5] highlighted the need for research to determine how patients and allied health professionals are responding to the initiative. Therefore, the aim of this study was to evaluate the utilisation of podiatry services under the EPC program between 2004 and 2008, by extracting data from the Medicare Benefits Schedule database [6]. Specifically, the total number of podiatry services provided compared to other allied health professions, sex and age differences in the number of services provided, trends over time, and total costs were explored.

## Methods

### Data extraction from the Medicare Benefits Schedule database

Data pertaining to all allied health professional item numbers under the EPC chronic disease management program for the calendar years 2004-2008 were extracted from the Medicare Benefits Schedule (MBS) database (item numbers 10950-10970) [6]. The complete dataset for item 10962 (consisting of the number of podiatry consultations provided according to sex, age-group, calendar year and state, along with matching cost data) was extracted and exported into Microsoft Excel (Microsoft Corp, Redmond USA) for analysis. To evaluate the trend in the total number of consultations per year between 2004 and 2008, per capita figures were extracted, as the number of people enrolled in Medicare each year varied over the assessment period. To evaluate the number of consultations provided in each state, both unadjusted and adjusted figures were calculated. Adjustments were based on state population data in the September 2008 quarter provided by the Australian Bureau of Statistics [7].

### Statistical analysis

Data were analysed using simple descriptive statistics (total number of consultations cross-tabulated by sex, age-group, calendar year and state), as the publicly accessible version of the MBS database does not allow for the extraction of individual-level data.

## Results

### Total number of consultations provided

**Figure 2:**
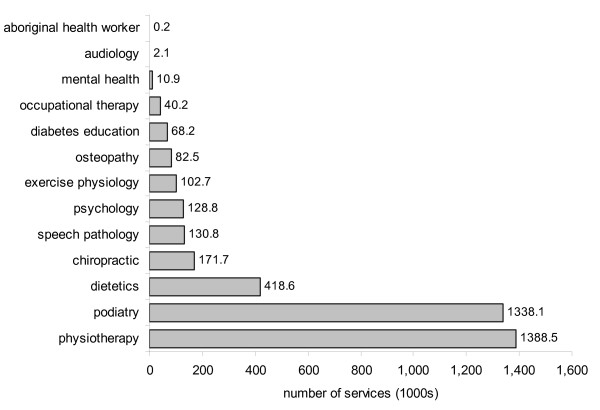
**Total number of EPC consultations between 2004 and 2008 for each allied health profession**.

The total number of EPC consultations provided for each of the allied health professions between 2004 and 2008 is shown in Figure 2. A total of 1,338,044 EPC services were provided by podiatrists, second only to physiotherapy (1,388,460 services). Podiatry services accounted for 34% of all EPC consultations provided by allied health professionals.

### Service provision by state

**Figure 3:**
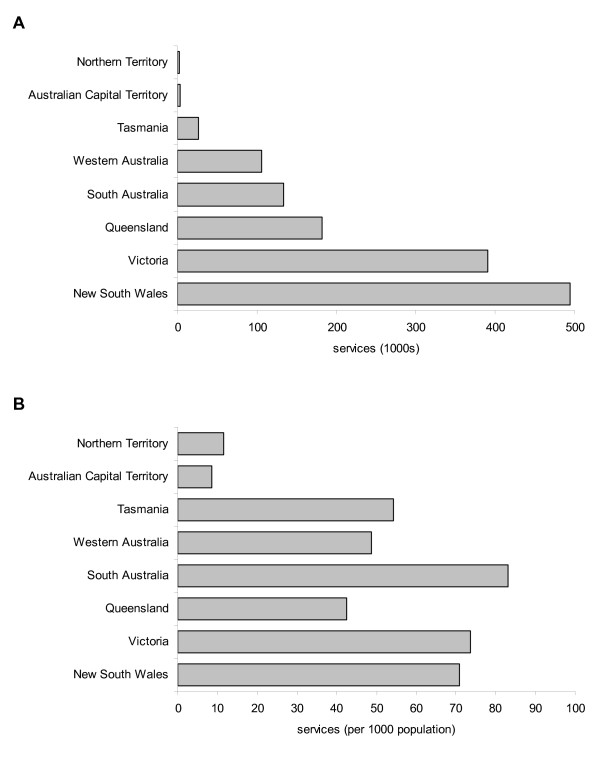
**Total (a) and per capita (b) podiatry EPC consultations between 2004 and 2008 by state**.

The total number of EPC podiatry consultations by state is shown in Figure 3. The highest proportion was provided in New South Wales (494,420, or 37%). However, when expressed relative to population, South Australia exhibited the highest proportion of EPC podiatry consultations (83 per 1,000 population).

### Service provision by sex and age

**Figure 4:**
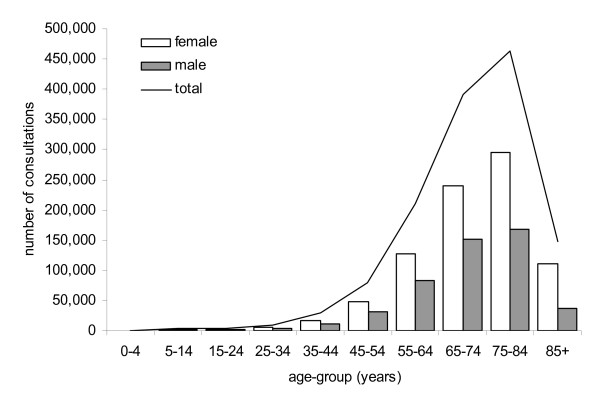
**Number of EPC podiatry consultations between 2004 and 2008 by sex and age**.

The total number of EPC podiatry consultations provided according to sex and age is shown in Figure 4. Females exhibited higher utilisation than males (63 versus 37%), and those aged over 65 years accounted for 75% of all consultations provided.

### Changes over time

**Figure 5:**
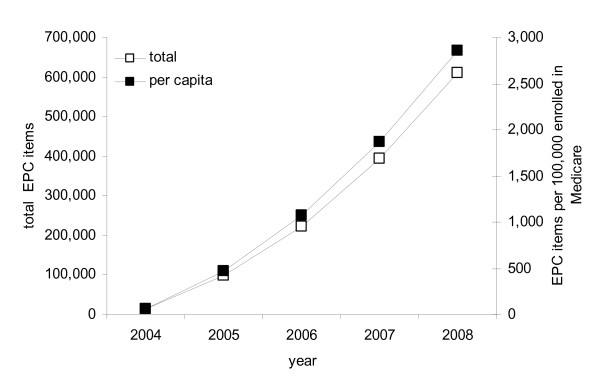
**Number of EPC podiatry consultations between 2004 and 2008, expressed as the total number of services and the number of services per 100,000 people enrolled in Medicare**.

The number of EPC podiatry consultations provided between 2004 and 2008 is shown in Figure 5, expressed as the total number of consultations and the number of consultations per 100,000 people enrolled in Medicare, as enrolment numbers fluctuate from year to year with births and deaths. There was a marked increase in the number of consultations over the five-year assessment period, both in absolute terms and relative to the number of people enrolled in Medicare.

### Costs

**Figure 6:**
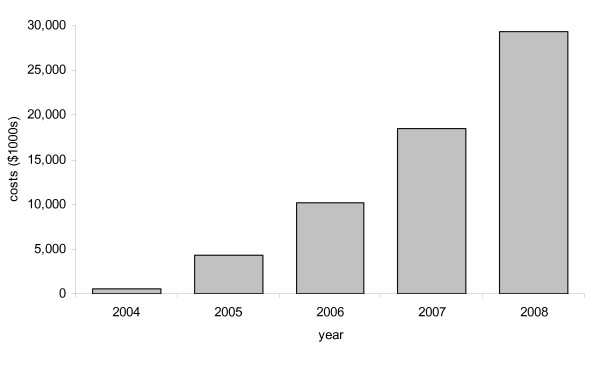
**Cost of subsidising EPC podiatry consultations per year between 2004 and 2008**.

The total cost of subsidising EPC podiatry consultations per year over the 2004-2008 period is shown in Figure 6. Over the five-year assessment period, the total cost was $62,888,196. This figure is slightly less than the number of consultations would indicate (which would be expected to be $65,497,254, i.e. the total number of consultations multiplied by $48.95), as cost data lags behind consultation data on the MBS database.

## Discussion

The objective of this study was to provide a basic descriptive analysis of podiatry services provided under the Australian Government's Enhanced Primary Care (EPC) chronic disease management program between 2004 and 2008. During this period, the analysis indicates that over 1.3 million EPC services were provided by podiatrists in Australia, which accounts for approximately one-third of the total number of allied health services provided by the program. This level of utilisation of podiatry services is striking when viewed in the context of the size of the podiatry labour force. The most recent labour force statistics estimate that in 2003, there were 1,820 practicing podiatrists in Australia - an extremely small number compared to other allied health professions such as physiotherapy (14,300), psychology (13,939) and occupational therapy (3,107) [8-11].

Utilisation of podiatry services was higher in women than men, and those aged over 65 years accounted for 75% of all services provided. The over-representation of women and those aged over 65 was expected, given that female sex [12-17] and age [12,16-19] are well-established risk factors for the development of foot problems. Furthermore, the findings are consistent with a recent South Australian study which reported that people who accessed podiatry services were more likely to be older, female, and have chronic conditions (such as obesity, osteoporosis, osteoarthritis, diabetes, cardiovascular disease and high blood pressure) [20]. Based on these findings, it would appear that EPC podiatry services are being accessed by population groups who have the greatest need for them.

Although the total number of consultations in each state was a simple reflection of population size, there was considerable variation when consultations in each state were expressed per capita, with South Australia having the highest rate of consultation (83 per 1,000 population). Several factors could be responsible for this variation between states, including population demographics (such as age and ethnicity) and socio-economic characteristics. It is interesting to note, however, that the per capita values are broadly reflective of each state's podiatry labour force. In 2003 (the most recent labour force data available), the number of full-time equivalent podiatrists per 100,000 population was as follows: South Australia - 17.4, Victoria - 13.0, Tasmania - 12.4, New South Wales: 9.3, and Queensland - 7.7 [8]. Although the public sector would absorb some of the demand for podiatry services, in most states over 75% of podiatrists work in the private sector. This suggests that the availability of private podiatry services in each state may play a role in determining the total number of consultations covered under the EPC scheme. If so, this may have equity implications for people in need of podiatry services residing in states with fewer podiatrists.

Between 2004 and 2008, there was a marked increase in the number of podiatry services provided under the EPC program, which reflects the overall trend in utilisation of the program by general practitioners and allied health professionals. The costs associated with the program have been substantial, with $62.9 M of government funding allocated for EPC podiatry rebates. However, it remains to be seen whether the present high growth in utilisation of EPC services is sustainable. Initial government estimates predicted a total cost of $247 M over the first four years [21]. However, over the 2004-2008 period, over six million rebates were provided, at a total cost of approximately half a billion dollars [6]. Although there have been anecdotal reports of inappropriate use of the program (including a $300 care plan involving a dietician and endocrinologist for a woman only 5 kg overweight [21]), no systematic audits of the EPC program have so far been conducted, although there are plans to do so.

Despite the apparent popularity of the EPC program with both health care providers and patients, several authors have expressed concerns regarding both its implementation and efficacy [5,22-25]. Focus group studies have indicated that many GPs consider the paperwork associated with the program to be excessive [26,27], and many believe the case conferencing item to be essentially impossible to implement [28]. Almost twice as many patients are being managed under GP management plans compared to team care arrangements, and it has been estimated that covering the cost of GP management plans accounts for approximately half of the cost of the entire program. Furthermore, an analysis of MBS data for 2007-2008 indicated that only a small proportion of patients on GP management plans were referred on for allied health services, and less than half of all GP management plans and only one-third of team care arrangements had been reviewed [22,24].

From the allied health professional's perspective, concerns have been raised that the care provided under the EPC program may be sub-optimal, as the number of funded treatments is often far less than what would normally be indicated in standard clinical practice [5]. The lack of remuneration of allied health professionals for non-treatment aspects of chronic disease management (such as report-writing and telephone contact with the GP) may also be significant disincentives to partake in the program [5,29]. Estimates from 2006 revealed that the average out-of-pocket expense for patients receiving an allied health service was $14 [22], which suggests that many allied health professionals, including podiatrists, are charging above the maximum rebate of $48.95 to cover their costs [30]. Given the relatively high overheads associated with podiatry service provision compared to other allied health services (due to factors such as the cost of consumables and instrument sterilisation), there is a sound argument for developing profession-specific rebates rather than a "one size fits all" fee schedule.

A modification to the EPC scheme to simplify allied health arrangements was announced by the health minister in January 2009, which specified that it is no longer necessary for a care planning item to have been claimed by the GP before allied health services can be provided [31]. This addressed the problem of allied health claims being rejected due to the GP plan having not yet been processed, despite the patient having a valid referral. The role of the GP as the "gate-keeper", however, is likely to remain a key feature of the program [31]. Whether this is the most appropriate model of service delivery is debateable. It could be reasonably argued that some of the funding currently allocated to cover GP management plans could be better utilised by funding more podiatry consultations under the EPC program, or by increasing funding for podiatry services in the public sector.

The data presented here need to be considered in the context of the inherent limitations of the MBS database. The database collates the number of *consultations *provided, rather than the number of *individuals *accessing allied health services, and as such, no accurate individual-level information can be extracted. Given that each patient is eligible for up to five allied health consultations per year, the actual number of individuals accessing podiatry services under the scheme during, for example, the 2008 calendar year, could be as low as 122,165 (i.e. the total number of consultations in that year divided by five), or as high as 610,829 (i.e. the total number of consultations in that year, assuming one consultation per patient). The actual number of patients accessing podiatry is likely to be somewhere in the middle of these lower and upper limits, but this cannot be accurately determined from the database. For the same reason, age and sex cross-tabulations may not provide an accurate estimate of the demographics of those accessing podiatry, as it is likely that older women are not only over-represented as patients, but also in terms of the number of consultations (i.e. older women may be more likely to be referred for, and "use up", all five of the allowable consultations than other population groups). Finally, the database does not collect information on the specific treatments provided during the consultation or comorbidities of those receiving podiatry services. While it is likely that a large proportion of consultations would involve general maintenance of nail and skin disorders and provision of foot care/footwear advice in people with diabetes, access to individual patient records would be required to confirm this.

Despite the inherent limitations of the MBS database, the data presented here clearly show that podiatry is a very significant component of the EPC program, and that subsidising podiatry services represents a major funding commitment by the Commonwealth government. Further research is required to assess whether the program improves health outcomes compared to standard clinical practice, and whether modifications to the scheme can improve accessibility, efficiency and cost-effectiveness.

## Conclusion

This analysis of the MBS database indicates that podiatry services have been extensively utilised under the EPC program by primary care patients, particularly older women. The number of podiatry services provided has increased dramatically between 2004 and 2008, which mirrors the escalating uptake of the program in general. Further research is required to determine whether the EPC program enhances clinical outcomes compared to standard practice, and whether modifications to the policy can improve the administration of the program.

## Competing interests

HBM is Editor-in-Chief of the *Journal of Foot and Ankle Research*. It is journal policy that editors are removed from the peer review and editorial decision making processes for papers they have authored or co-authored.

## References

[B1] Australian Institute of Health and Welfare (2006). Chronic disease and associated risk factors in Australia (AIHW cat no PHE 81) Canberra.

[B2] New Medicare rebate to encourage expansion of GP Care. http://www.health.gov.au/internet/main/publishing.nsf/Content/health-mediarel-yr1999-mw-mw99105.htm.

[B3] Expanded Medicare services for the chronically ill. http://www.health.gov.au/internet/ministers/publishing.nsf/Content/health-mediarel-yr2004-ta-abb092.htm.

[B4] Allied Health Services Under Medicare - Fact Sheet. http://www.health.gov.au/internet/main/publishing.nsf/Content/health-medicare-health_pro-gp-pdf-allied-cnt.htm.

[B5] Foster MM, Mitchell G, Haines T, Tweedy S, Cornwell P, Fleming J (2008). Does Enhanced Primary Care enhance primary care? Policy-induced dilemmas for allied health professionals. Med J Aust.

[B6] Medicare Benefits Schedule database. http://www.medicareaustralia.gov.au/provider/medicare/mbs.shtml.

[B7] Australian Bureau of Statistics (2009). Australian Demographic Statistics, Sep 2008 (31010).

[B8] Australian Institute of Health and Welfare (2006). Podiatry Labour Force 2003.

[B9] Australian Institute of Health and Welfare (2006). Physiotherapy Labour Force 2002.

[B10] Australian Institute of Health and Welfare (2006). Psychology Labour Force 2003.

[B11] Australian Institute of Health and Welfare (2006). Occupational Therapy Labour Force 2002-2003.

[B12] Garrow AP, Silman AJ, Macfarlane GJ (2004). The Cheshire Foot Pain and Disability Survey: a population survey assessing prevalence and associations. Pain.

[B13] Benvenuti F, Ferrucci L, Guralnik JM, Gangemi S, Baroni A (1995). Foot pain and disability in older persons: an epidemiologic survey. J Am Geriatr Soc.

[B14] Gorter KJ, Kuyvenhoven MM, deMelker RA (2000). Nontraumatic foot complaints in older people. A population-based survey of risk factors, mobility, and well-being. J Am Podiatr Med Assoc.

[B15] Menz HB, Morris ME (2005). Determinants of disabling foot pain in retirement village residents. J Am Podiatr Med Assoc.

[B16] Hill CL, Gill T, Menz HB, Taylor AW (2008). Prevalence and correlates of foot pain in a population-based study: the North West Adelaide Health Study. J Foot Ankle Res.

[B17] Farndon L, Barnes A, Littlewood K, Harle J, Beecroft C, Burnside J, Wheeler T, Morris S, Walters SJ (2009). Clinical audit of core podiatry treatment in the NHS. J Foot Ankle Res.

[B18] Brodie BS, Rees CL, Robins DJ, Wilson AFJ (1988). Wessex Feet: a regional foot health survey, Volume I: The survey. Chiropodist.

[B19] Greenberg L, Davis H (1993). Foot problems in the US. The 1990 National Health Interview Survey. J Am Podiatr Med Assoc.

[B20] Menz HB, Gill TK, Taylor AW, Hill CL (2008). Predictors of podiatry utilisation in Australia: the North West Adelaide Health Study. J Foot Ankle Res.

[B21] Anastopoulos C (2006). Chronic disease items blow budget. Australian Doctor.

[B22] Russell LM (2008). A primary care reform agenda for Australia.

[B23] Swerissen H, Taylor MJ (2008). Reforming funding for chronic illness: Medicare-CDM. Aust Health Rev.

[B24] Russell L (2009). Reform must improve quality of life.

[B25] Hartigan PA, Soo TM, Kljakovic M (2009). Do team care arrangements address the real issues in the management of chronic disease?. Med J Aust.

[B26] Wilkinson D, McElroy H, Beilby J, Mott K, Price K, Morey S, Best J (2002). Uptake of health assessments, care plans and case conferences by general practitioners through the Enhanced Primary Care program between November 1999 and October 2001. Aust Health Rev.

[B27] Oldroyd J, Proudfoot J, Infante FA, Davies GP, Bubner T, Holton C, Beilby JJ, Harris MF (2003). Providing healthcare for people with chronic illness: the views of Australian GPs. Med J Aust.

[B28] Mitchell GK, DeJong IC, DelMar CB, Clavarino AM, Kennedy R (2002). General practitioner attitudes to case conferences: how can we increase participation and effectiveness?. Med J Aust.

[B29] Lewis P, White A, Misan G, Harvey P, Connolly J, Noone J (2003). Enhanced primary care. A rural perspective. Aust Fam Physician.

[B30] Do you bulk-bill on podiatry Medicare items?. http://www.podiatry-arena.com/podiatry-forum/showthread.php?t=1442.

[B31] Enhanced Primary Care Program (EPC): simpler administrative arrangements for allied health. http://www.health.gov.au/internet/main/publishing.nsf/Content/admin-arrangement-for-allied-health.

